# Degradation of Polyester Polyurethane by Bacterial Polyester Hydrolases

**DOI:** 10.3390/polym9020065

**Published:** 2017-02-16

**Authors:** Juliane Schmidt, Ren Wei, Thorsten Oeser, Lukas Andre Dedavid e Silva, Daniel Breite, Agnes Schulze, Wolfgang Zimmermann

**Affiliations:** 1 Department of Microbiology and Bioprocess Technology, Institute of Biochemistry, Leipzig University, 04103 Leipzig, Germany; juliane.schmidt@uni-leipzig.de (J.S.); t.oeser@web.de (T.O.); lucasandred@gmail.com (L.A.D.S.); 2 Leibniz Institute of Surface Modification, Permoserstr. 15, 04318 Leipzig, Germany; daniel.breite@iom-leipzig.de (D.B.); agnes.schulze@iom-leipzig.de (A.S.)

**Keywords:** polyester polyurethane, enzymatic hydrolysis, polyester hydrolases

## Abstract

Polyurethanes (PU) are widely used synthetic polymers. The growing amount of PU used industrially has resulted in a worldwide increase of plastic wastes. The related environmental pollution as well as the limited availability of the raw materials based on petrochemicals requires novel solutions for their efficient degradation and recycling. The degradation of the polyester PU Impranil DLN by the polyester hydrolases LC cutinase (LCC), TfCut2, Tcur1278 and Tcur0390 was analyzed using a turbidimetric assay. The highest hydrolysis rates were obtained with TfCut2 and Tcur0390. TfCut2 also showed a significantly higher substrate affinity for Impranil DLN than the other three enzymes, indicated by a higher adsorption constant *K*. Significant weight losses of the solid thermoplastic polyester PU (TPU) Elastollan B85A-10 and C85A-10 were detected as a result of the enzymatic degradation by all four polyester hydrolases. Within a reaction time of 200 h at 70 °C, LCC caused weight losses of up to 4.9% and 4.1% of Elastollan B85A-10 and C85A-10, respectively. Gel permeation chromatography confirmed a preferential degradation of the larger polymer chains. Scanning electron microscopy revealed cracks at the surface of the TPU cubes as a result of enzymatic surface erosion. Analysis by Fourier transform infrared spectroscopy indicated that the observed weight losses were a result of the cleavage of ester bonds of the polyester TPU.

## 1. Introduction

Polyurethanes (PU) are synthetic polymers present in many aspects of our daily life. Otto Bayer was the first to produce PU in 1937 by a polymerization reaction of di- or polyisocyanates and polyols [1]. By varying the nature of the components as well as the degree of cross-linking, PU materials with almost any desired properties can be synthesized [1]. For example, polyester or polyether polyurethanes can be synthesized by using polyester or polyether resins that contain hydroxyl groups [2]. PU is used in many industrial applications to produce foams, elastomers, coatings, adhesives and sealants applied in automotive, furniture, bedding, textiles, and other industrial areas [3]. The demand for PU in Europe amounted to 3.4 million tons in 2013 [4]. The large amounts of synthetic polymers produced has resulted in a massive increase of plastic waste requiring novel recycling strategies.

Both fungi [5,6,7,8] and bacteria [9,10,11,12,13,14,15,16,17] have been reported to degrade PU materials. Darby and Kaplan were the first to test the susceptibility of different PU materials against fungal attack [7]. All polyester PU were found to be highly susceptible to degradation by *Aspergillus*, *Penicillium*, *Pullularia*, *Trichoderma* and *Chaetomium* species whereas polyether PU showed a higher resistance. The fungal strain PURDK2 has been reported to degrade urethane and urea bonds also in polyether PU [18]. Recently, eight further fungal strains isolated from environmental samples have been shown to degrade both polyester and polyether PU [19].

Among bacteria, *Corynebacterium* species, *Enterobacter agglomerans* and *Bacillus subtilis* species can efficiently degrade PU samples [12,16]. An esterase from *Comamonas acidovorans* TB-35 was reported as the responsible enzyme for the hydrolysis of the ester bonds in a polyester PU [20]. A polyamidase from *Nocardia farcinica* fused to a polymer binding module of a polyhydroxyalkanoate depolymerase from *Alcaligenes faecalis* has been reported to cleave the urethane bond in PU polyesters with different degrees of crystallinity [21].

Enzymes capable of hydrolyzing synthetic polyesters are produced by various moderate thermophilic actinomycetes, including *Thermobifida alba* AHK119 [22,23], *Thermobifida fusca* [24,25,26,27,28], *Thermomonospora curvata* DSM43183 [29] and *Saccharomonospora viridis* AHK 190 [30,31]. Recently, the polyester hydrolase LC cutinase (LCC) was identified using a metagenomic library derived from plant compost [32,33]. Polyester hydrolases from actinomycetes belong to the α/β hydrolase fold superfamily [25,34,35] and have catalytic properties between lipases and esterases [36]. The polyester hydrolases TfCut2, Tcur0390 and Tcur1278 isolated from *Thermobifida fusca* KW3 and *Thermomonospora curvata* DSM43183, respectively [35], as well as LCC have previously been shown to hydrolyze the synthetic aromatic polyester polyethylene terephthalate (PET) [26,29,32].

In this study we show that polyester hydrolases from actinomycetes are able to degrade both the anionic aliphatic polyester PU dispersion Impranil DLN and the thermoplastic polyester PU (TPU) Elastollan B85A-10 and C85A-10.

## 2. Materials and Methods

### 2.1. Genes, Enzymes and Chemicals

The synthetic gene constructs for LCC, Tcur0390 and Tcur1278 with adapted codon usage for *Escherichia coli* were synthesized by GeneArt Gene Synthesis (Life Technologies GmbH, Darmstadt, Germany). Impranil DLN, an aqueous polyester PU dispersion with a solids content of approx. 40% was kindly supplied by Bayer MaterialScience AG (Leverkusen, Germany). The highly pure TPU Elastollan B85A-10 and Elastollan C85A-10 without additives were kindly supplied by BASF Polyurethanes GmbH (Lemfoerde, Germany). All other chemicals were obtained from Carl Roth GmbH + Co. KG (Karlsruhe, Germany) at highest purity available.

### 2.2. Cloning, Expression and Agar Plate Screening of the Polyester Hydrolase Genes

The synthetic gene constructs encoding Tcur0390 and Tcur1278 without the secretion signal peptides were amplified using the primers Tcur0390-FW (5′-TTTT GGA TCC G GCA AAT CCG TAT CAG CGT GG-3′), Tcur0390-RV (5′-TTTT GAA TTC CC CAT CGG ACA GGT AAA ACG TGC-3′), Tcur1278-FW (5′-TTTT GGA TCC G GCA AAT CCG TAT CAG CGT GG-3′) and Tcur1278-RV (5′-TTTT GAA TTC CC GGT ATG CGG ACA GGT ATC AC-3′) (the restriction sites for *Bam*HI and *Eco*RI are underlined). The amplification of the genes encoding for TfCut2 and LCC as well as the cloning of all recombinant genes was performed as described before [37]. Transformants expressing active hydrolases were identified on turbid lysogeny broth (LB) agar plates containing 100 µg/mL ampicillin, 0.4 mM isopropyl β-d-1-thiogalactopyranoside (IPTG) and 0.5% Impranil DLN. The expression and purification of the recombinant enzymes was done as described before [38]. Amicon Ultra Centrifugal Filter Units (Merck KGaA, Darmstadt, Germany) were used to concentrate the purified enzymes and change the buffer to 10 mM Tris-HCl (pH 8.0).

### 2.3. Turbidimetric Assay Using Impranil DLN Dispersion

Enzymatic hydrolysis of Impranil DLN dispersion (40%) was investigated by a turbidimetric assay. The samples with a final volume of 250 µL were prepared in 96 well plates containing 0.3 M potassium phosphate buffer (pH 8.0) and different amounts of the purified hydrolases. The reaction was started by adding Impranil DLN at a final concentration of 0.1%. The decrease of the turbidity of the samples was monitored by measuring the optical density at 400 nm (OD_400_) at constant time intervals of 7 s at room temperature. The hydrolysis rates were determined from the linear part of the graphs of the decreasing OD_400_ over time. All determinations were performed at least in duplicate. The rate constant of the surface reaction *k*_s_ as well as the adsorption equilibrium constant *K* were determined by a kinetic model proposed by Mukai et al. [39]:

(1) $$R=\frac{k_{s}K\left[E\right]}{{\left(1+K\left[E\right]\right)}^{2}}$$

where [*E*] is the enzyme concentration and *R* is the hydrolysis rate. This equation is converted to its linear form:

(2) $${\left(\frac{\left[E\right]}{R}\right)}^{\frac{1}{2}}=\frac{K\left[E\right]}{\alpha }+\frac{1}{\alpha }$$

$$\alpha ={\left(k_{s}K\right)}^{\frac{1}{2}}$$

### 2.4. Degradation of Solid Polyester TPU Cubes

For the determination of the weight loss of TPU Elastollan B85A-10 and Elastollan C85A-10 following enzymatic degradation, TPU cubes (0.5 cm × 0.5 cm × 0.2 cm, about 80 mg) were added to reaction vials containing 50 µg of purified enzyme and 1 M potassium phosphate buffer (pH 8.0) in a total volume of 1 mL. The vials were incubated at 60–70 °C on a thermo shaker (1000 rpm) for 100–200 h. The TPU degradation was quantified by measuring the weight loss of the TPU cubes. All measurements were made at least in triplicate.

### 2.5. Scanning Electron Microscopy

The Elastollan surface was investigated by scanning electron microscopy (SEM, Ultra 55, Carl Zeiss SMT, Jena, Germany). The samples were preliminarily coated with a 50 nm layer of chrome to improve the conductivity.

### 2.6. Fourier Transform Infrared Spectroscopy

Fourier transform infrared spectroscopy (FTIR) measurements were performed in ATR (attenuated total reflection) mode with the FTIR spectrometer Vector 22 (Bruker Corporation, Billerica, MA, USA) and a diamond crystal (Specac, Kent, UK). With ATR-IR, only the outer surface of the samples is investigated. Changes in the IR spectra indicate a surface erosion process caused by the enzymes. The spectra were obtained in the wavenumber range of 550–4000 cm^−1^ using a spectral resolution of 2 cm^−1^.

### 2.7. Gel Permeation Chromatography

Gel permeation chromatography (GPC) of the TPU Elastollan B85A-10 and Elastollan C85A-10 were conducted using dimethylformamide (DMF) as eluent on a Waters GPC system (Waters Co., Milford, CT, USA) consisting of a Waters 1515 isocratic HPLC pump, a Waters 2414 refractive index detector, and Waters 717 autosampler. The system was equipped with three columns containing polyester copolymer particles (10 µm particle size, 1000 Å/100 Å/30 Å, 4.6 mm × 250 mm, PSS Polymer Standards Service GmbH, Mainz, Germany). The flow rate was set to 0.33 mL/min. The resulting data was evaluated using the Breeze software (Dallas, TX, USA).

## 3. Results

### 3.1. Hydrolysis of Impranil DLN by the Polyester Hydrolases

Cultures of *E. coli* expressing TfCut2, LCC, Tcur0390 and Tcur1278 formed halos on agar plates containing 0.5% Impranil DLN, indicating PU-degrading activity.

**Figure 1:**
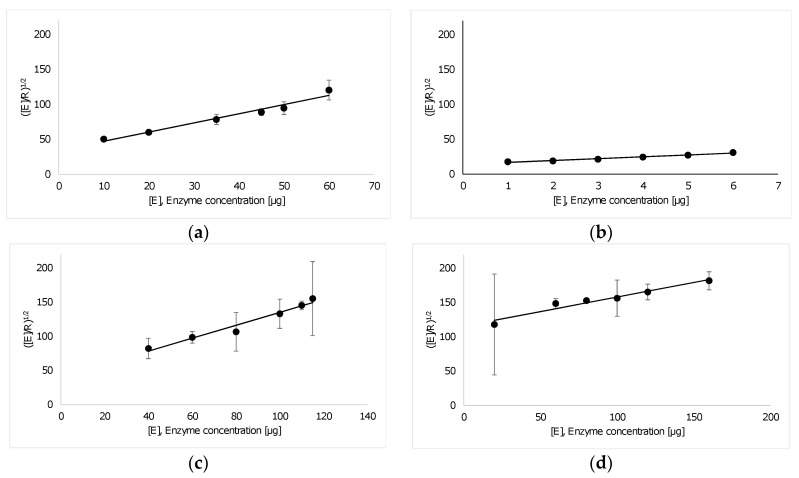
Linear plots of ${\left(\frac{\left[E\right]}{R}\right)}^{\frac{1}{2}}$ (circles) of Impranil DLN hydrolysis as a function of the concentration of (**a**) LCC; (**b**) TfCut2; (**c**) Tcur0390; and (**d**) Tcur1278. The fitting data (solid lines) are based on the kinetic model proposed by Mukai et al. [39] (Equation (2)). Error bars indicate the standard deviation of duplicate determinations.

The kinetics of enzymatic degradation of a 0.1% Impranil DLN was analyzed using a turbidimetric assay. The OD_400_ of the substrate dispersion revealed a linear decrease in the initial reaction phase and then leveled off to 30%–40% of its initial value during degradation with all enzymes (data not shown). The initial hydrolysis rates were determined from the linear part of the decreasing OD_400_ values over the reaction time and were used to calculate the kinetic parameter as shown in Figure 1.

TfCut2 and Tcur0390 showed the highest hydrolysis rate constants of 0.026 s^−1^ for Impranil DLN (Table 1). The lowest enzyme concentration required to reach the maximum initial hydrolysis rate was obtained with TfCut2. The highest adsorption constant *K* was obtained with TfCut2, indicating its strong affinity to Impranil DLN.

### 3.2. Degradation of Solid Polyester TPU Cubes

**Figure 2:**
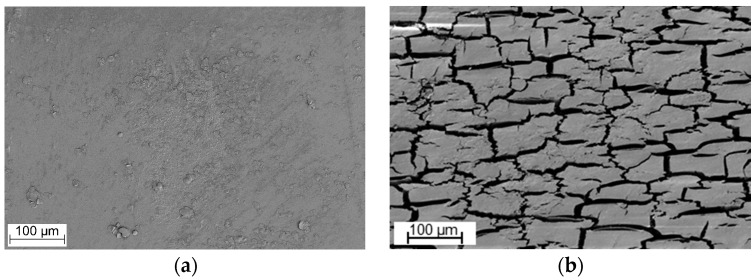

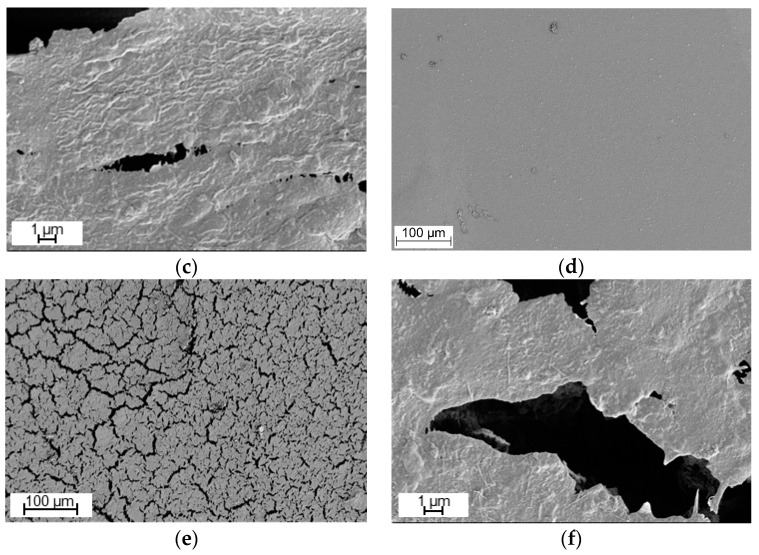
SEM images of TPU cube surfaces: (**a**–**c**) Elastollan B85A-10; and (**d**–**f**) Elastollan C85A-10; (**a**,**d**) negative control samples without enzyme; and (**b**,**c**,**e**,**f**) following hydrolysis by LCC at 70 °C for 200 h.

**Figure 3:**
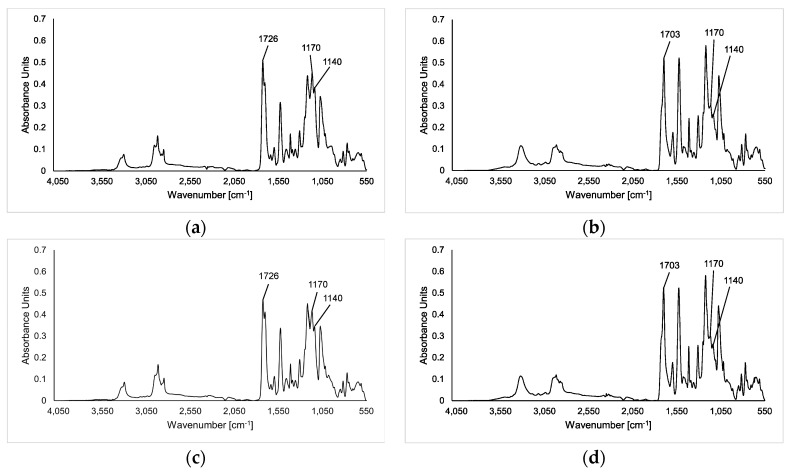
FTIR spectra of (**a**,**b**) Elastollan B85A-10 and (**c**,**d**) Elastollan C85A-10: (**a**,**c**) negative control samples without enzyme and (**b**,**d**) following hydrolysis by TfCut2 at 60 °C for 100 h.

Table 2 shows the weight loss of Elastollan B85A-10 and C85A-10 determined after enzymatic degradation at 60–70 °C for 100 h. Due to their lower thermal stability [29], Tcur0390 and Tcur1278 were not used for TPU degradation experiments at 70 °C. No significant weight loss was detected with negative control samples in the absence of enzymes. At 60 °C, a weight loss of about 1% was obtained with TfCut2 and LCC, whereas Tcur0390 and Tcur1278 caused only a minor weight reduction of the TPU cubes. At 70 °C, LCC caused weight losses of up to 3.2% which were significantly higher than those obtained with TfCut2. The better performance of LCC at 70 °C was likely the result of its higher thermostability compared to TfCut2 [35]. Elastollan B85A-10 appeared also to be more easily degradable by enzymes than C85A-10. Extending the reaction time to 200 h, LCC caused weight losses of 4.9% and 4.1% for Elastollan B85A-10 and C85A-10 at 70 °C, respectively. An analysis by SEM revealed large cracks at the surface of both solid TPU samples after a reaction time of 200 h with LCC (Figure 2). The Elastollan B85A-10 and C85A-10 samples were also analyzed by FTIR spectroscopy before and after hydrolysis by TfCut2 (Figure 3). The shift of the absorption peak at around 1726 to 1703 cm^−1^ indicated a change of the carbonyl group in the polyester components of TPU and thus the cleavage of ester bonds [40,41]. A reduction of the peaks at about 1170 and 1140 cm^−1^ which represent a C–O stretching also confirmed the change of the ester components of TPU [16]. Spectra obtained by FTIR spectroscopy following hydrolysis by LCC at 70 °C did not differ significantly from those obtained with TPU samples treated at 60 °C, indicating that the observed weight losses were in all cases a result of the cleavage of ester bonds, independent of the reaction temperature and the enzyme used.

GPC analysis revealed a significant decrease of up to 19% of the weight-averaged molecular weights (*M*_w_) and of up to 21% of the peak molecular weights (*M*_p_) of Elastollan B85A-10 and C85A-10 hydrolyzed by LCC compared to the negative control samples without enzyme (Table 3). The number-averaged molecular weight (*M*_n_) of both Elsatollans did not change following incubations with the enzymes since the large proportion of bulk polymer inside the TPU cubes was not exposed to an enzymatic hydrolysis. The changes in *M*_w_ of the solid TPU samples indicated a preferential degradation of the longer polymer chains by the polyester hydrolase at the polymer surface.

## 4. Discussion

In this study, the polyester hydrolases TfCut2, Tcur0390, Tcur1278 and LCC were shown to degrade both dispersed and solid polyester PU materials. Impranil DLN is an aqueous aliphatic polyester PU dispersion made from a poly hexane/neopentyl adipate polyester and hexamethylene diisocyanate. It has been widely used as a substrate to detect PU-hydrolyzing activity of microbial strains. The recombinant *E. coli* colonies expressing the four polyester hydrolases produced clearing zones in agar media containing Impranil DLN. This PU substrate can also be used to detect enzymatic PU-degrading activity [9,10,42]. The PU degradation by fungal strains has been previously quantified by monitoring the change of the optical density of Impranil DLN dispersions at 600 nm following an incubation with the strains [19]. In this study, we developed a turbidimetric assay based on Impranil DLN to analyze the kinetics of enzymatic PU hydrolysis. Rapid PU degradation was observed with all four hydrolases as indicated by the decrease of OD_400_ in the first minutes of the reaction. In contrast to a similar assay developed for monitoring the enzymatic degradation of PET nanoparticle suspensions [43], Impranil DLN did not agglutinate in the presence of buffered enzyme solutions. It was therefore not necessary to immobilize the dispersed PU particles during the enzymatic reaction which enabled a high-throughput determination of the PU-hydrolyzing activity in microplate format.

For the hydrolysis of Impranil DLN, TfCut2 and Tcur0390 revealed the highest rate constants (Table 1). In contrast, Tcur0390 caused a significantly lower degradation performance of the Elastollan TPU cubes (Table 2). Increasing the amount of Tcur0390 did not result in a further degradation of the TPU cubes, suggesting that the enzymatic hydrolysis of Impranil DLN and Elastollan followed distinct kinetics due to their different composition. The dispersed PU Impranil DLN provided a larger specific surface area resulting in a better accessibility for the enzymes than the solid TPU materials. Indeed, the enzymatic hydrolysis of Impranil DLN was dependent on the enzyme concentration applied, especially Tcur0390, which showed a significantly lower substrate affinity than TfCut2 (Table 1).

Several publications have reported weight losses of PU materials after incubation with fungal or bacterial strains. A weight loss of a polyester PU foam was observed after incubation with *Gliocladium roseum* for 21 days [44] and weight losses of up to 17.7% were detected after incubation of this material with 16 bacterial strains for 12 weeks [12]. The fungal strain *Cladosporium tenuissimum* A2.PP.5 caused a weight loss of 65.3% from a polyether PU foam after incubation for 21 days at 25–30 °C [19]. In these studies, microorganisms were incubated for several weeks with the PU foams which provided a large surface area compared to the solid cubes of Elastollan used here, explaining the higher weight losses observed from the former PU material. In contrast, the enzymatic hydrolysis of the Elastollan cubes occurred solely at the surface of the material, as indicated by the cracks detected by SEM (Figure 2).

*C. acidovorans* TB-35 completely degraded polyester PU cubes of about 50 mg within seven days of incubation in a liquid medium supplemented with PU as the sole carbon source [45]. A cell-surface bound and a secreted esterase were isolated from this strain. The crude enzyme solution caused a weight loss of polyester PU cubes of 13% after a reaction time of 48 h at 30 °C [46]. Similar absolute weight losses of the Elastollan cubes were obtained with TfCut2 and LCC at 60 °C. Hydrolysis by LCC for 200 h at 70 °C resulted in the weight loss of 4.9% and 4.1% for Elastollan B85A-10 and C85A-10, respectively. TfCut2 caused only slightly higher weight losses at a reaction temperature of 70 °C (Table 2). This may be due to the lower thermal stability of TfCut2 compared to LCC [33,35]. GPC analysis of the Elastollan samples hydrolyzed by LCC showed a preferred degradation of the longer polymer chains indicated by a significant decrease of their *M*_w_ of about 20%.

The FTIR analysis of the enzymatically treated polyester TPU cubes indicated a cleavage of ester bonds in both Elastollan samples while the urethane bonds resisted the enzymatic hydrolysis (Figure 3). This result confirms previous reports which claimed that the esterase-catalyzed hydrolysis of polyester PU was mainly a result of the cleavage of ester bonds in the polyester polyol but not of the urethane bonds [16,20]. A turbidity of 30%–40% of the Impranil DLN dispersions remaining after enzymatic hydrolysis also indicated that the PU was only partially degraded by the polyester hydrolases.

In conclusion, we demonstrated the hydrolysis of both polyester PU dispersions and thermoplastic polyester PU cubes by the polyester hydrolases TfCut2, LCC, Tcur0390 and Tcur1278. A turbidimetric assay was validated for the kinetic analysis of the enzymatic degradation of Impranil DLN. While most studies have reported reaction temperatures for the enzymatic hydrolysis of PU of less than 40 °C [19,45,46], reaction temperatures of up to 70 °C were shown to significantly increase the enzymatic degradation of solid TPU materials. Hence, thermostable polyester hydrolases proved to be promising catalysts for the hydrolysis of solid polyester PU with potential applications in PU recycling processes.

## Figures and Tables

**Table 1:** Kinetic parameters of the enzymatic hydrolysis of Impranil DLN determined by the kinetic model proposed by Mukai et al. [39] (Equation (2)).

Enzyme	*K* (µg^−1^)	*k^s^* (s^−1^)	*R*^2^
TfCut2	0.188 ± 0.024	0.026 ± 0.001	0.972
LCC	0.038 ± 0.009	0.022 ± 0.002	0.955
Tcur0390	0.023 ± 0.007	0.026 ± 0.003	0.954
Tcur1278	0.004 ± 0.001	0.020 ± 0.002	0.938

**Table 2:** Weight loss of TPU Elastollan B85A-10 and C85A-10 cubes with an initial weight of approximately 80 mg after hydrolysis by LCC, TfCut2, Tcur0390 and Tcur1278 for 100 h at 60–70 °C. All values were determined at least in triplicate.

Enzyme	Temperature (°C)	Weight loss (%)	
		B85A-10	C85A-10
LCC	60	1.2 ± 0.2	1.2 ± 0.2
	70	3.2 ± 0.5	2.5 ± 0.4
TfCut2	60	1.0 ± 0.1	1.1 ± 0.2
	70	1.9 ± 0.3	1.5 ± 0.2
Tcur0390	60	0.3 ± 0.1	0.4 ± 0.0
Tcur1278	60	0.6 ± 0.1	0.8 ± 0.1

**Table 3:** Molecular weights of Elastollan B85A-10 and C85A-10 determined by GPC following hydrolysis by LCC at 70 °C for 200 h and of negative control samples without enzyme. The polydispersity index (PDI) is the quotient of *M*_w_ and *M*_n_.

Elastollan	Weight loss (%)	*M*_n_	*M*_w_	*M*_p_	PDI
B85A-10	0 (negative control)	57,355 ± 1284	132,425 ± 1948	97,559 ± 1011	2.31
	4.9	57,635 ± 891	108,873 ± 2688	77,501 ± 3176	1.89
C85A-10	0 (negative control)	49,061 ± 1514	109,264 ± 1024	73,335 ± 3426	2.23
	4.1	49,009 ± 1613	88,956 ± 1530	66,288 ± 2846	1.82
